# Development of two compatible plasmids to assess sRNA-mediated post-transcriptional regulation in Acinetobacter baumannii

**DOI:** 10.1099/mic.0.001639

**Published:** 2025-11-25

**Authors:** Aalap Mogre, Orla Connell, Jessica White, Ali Shaibah, Karsten Hokamp, Fergal J. Hamrock, Kristina Schauer, Carsten Kröger

**Affiliations:** 1 Department of Microbiology School of Genetics & Microbiology, Moyne Institute of Preventive Medicine, Trinity College Dublin, Dublin, Ireland; 2 Department of Biological Science, Faculty of Science, King Abdulaziz University, Jeddah, Saudi Arabia; 3 Department of Genetics, School of Genetics & Microbiology, Smurfit Institute of Genetics, Trinity College Dublin, Dublin, Ireland

**Keywords:** *Acinetobacter baumannii*, post-transcriptional regulation, small RNAs, two-plasmid system

## Abstract

Post-transcriptional regulation can be mediated by small regulatory RNAs (sRNAs) in bacteria, which can act by base-pairing to a target mRNA. The discovery and mechanistic validation of base-pairing sRNAs in multidrug-resistant *Acinetobacter baumannii* has been hampered by the lack of genetic tools to assess RNA–RNA interactions. Here, we created two compatible plasmids for *A. baumannii*, which addresses this need. The newly designed plasmids validated the known Aar sRNA-*carO* mRNA and a new interaction of sRNA44 and the mRNA of the biofilm-associated protein Bap. The new plasmid system should accelerate the mechanistic characterization of sRNAs in *A. baumannii*.

**Impact Statement**

Multidrug resistance of pathogenic micro-organisms is one of the greatest challenges for modern medicine. Carbapenem-resistant *Acinetobacter baumannii* is considered a highly critical organism, yet we are only beginning to understand its physiology and mechanisms of gene regulation. Post-transcriptional regulation by base-pairing small RNAs is an understudied area, partly because of the lack of genetic tools to investigate them. In this study, we developed a two-plasmid system to assess sRNA–mRNA interactions, which will greatly accelerate the discovery and validation of small regulatory RNAs and their target molecules.

## Data Summary

Plasmid sequences of pAMCK14-sRNA44 and pAMCK18-Bap have been made available in GenBank of National Center for Biotechnology Information (accession numbers PV916437 and PV916438).

## Introduction

Small regulatory RNAs (sRNAs) are ubiquitous post-transcriptional regulators in bacteria [1,2]. Many of them function by base-pairing to another RNA molecule, typically an mRNA molecule, to modulate its translation [3]. One of the challenges in determining the biological function of base-pairing regulatory sRNAs is identifying their target mRNA. Methods to identify RNA–RNA interactions range from bioinformatic predictions to several experimental approaches that ideally should be combined [4]. Several methods have been developed to experimentally verify RNA–RNA interactions, including high-throughput, proximity ligation-based methods such as RIL-seq, Hi-GRIL-seq, iRIL-seq or CLASH [5,6]. As these methods rely on proximity ligation, which can ligate transiently interacting RNAs that do not represent biologically relevant RNA–RNA pairs, RNA–RNA interactions identified by high-throughput methods must be independently validated to distinguish biologically relevant from transient, less relevant interactions.

A genetic system consisting of two replication-compatible plasmids has been successfully used to validate and dissect mechanistic details of sRNA–mRNA interactions in *Enterobacteriaceae* [7,9]. The sRNA is expressed from one plasmid and is assessed whether it alters the translation of the target mRNA-GFP (or superfolder GFP) reporter gene fusion expressed from the second plasmid [7,10, 11]. Introduction of nucleotide changes to disrupt and restore interacting sRNA–mRNA duplexes allows precise identification of nucleotides essential for base-pairing. The original two-plasmid system has been instrumental in characterizing many regulatory sRNAs in *Escherichia coli* and *Salmonella enterica*. However, these original plasmids are not always compatible with (co-)replication and selection in other Gram-negative bacteria, hindering the progress of studying sRNA-mediated post-transcriptional regulation in non-model or multidrug-resistant organisms. For example, these plasmids are not replicative in * A. baumannii*, where much less is known about the regulatory functions and biological roles of sRNAs [12].

Recently, it was demonstrated that plasmids harbouring the pWH1266 and pRSF1010 origins of replication can be stably co-maintained in *Acinetobacter* [13,14]. Arguably, the most used plasmid in *A. baumannii* is pWH1266, which is a shuttle plasmid constructed from the fusion of a natural *Acinetobacter calcoaceticus* plasmid pWH1277 and pBR322 [15]. The pWH1266 origin of replication was subsequently widely used to generate plasmids for gene overexpression or chromosomal integration in *A. baumannii* under the control of various regulatory systems [14,16,19]. Plasmids with a pRSF1010 origin of replication support a broad host range and were shown to replicate in *Acinetobacter* spp. decades ago [20]. To create a two-plasmid system for assessing sRNA–mRNA interactions in *A. baumannii*, we aimed to generate two plasmids carrying the replication-compatible pWH1266- and pRSF1010-derived origin of replications. The plasmids were evaluated for their ability to replicate and functionally substitute for the historical pP_L_- and pXG-plasmids created for *Enterobacteriaceae* using the same constitutive P_LtetO_ and P_LlacO_ promoters and a truncated sfGFP to create translational fusions in *A. baumannii* [7,10, 11].

## Methods

### Bacterial strains, growth conditions and transformations

*E. coli* TOP10 and *A. baumannii* AB5075 were maintained on lysogeny (l-) broth agar plates (Lennox, 10 g l^−1^ tryptone, 5 g l^−1^ yeast extract, 5 g l^−1^ NaCl, 15 g l^−1^ agar) at 37 °C. Antibiotics [tetracycline (12 µg ml^−1^) and apramycin (60 µg ml^−1^)] were added when required. *E. coli* cells were transformed by electroporation or heat shock transformation. *A. baumannii* AB5075 cells were transformed by electroporation.

### Chemical transformation

*E. coli* TOP10 cells were made chemically competent using the transformation and storage solution (TSS) method [21]. A 4-ml overnight culture was set up from a single colony and incubated at 37 °C, 220 r.p.m. The next day, the culture was diluted 1:100 in 50 ml l-broth in a 250 ml Erlenmeyer flask. The flask was incubated at 37 °C, 220 r.p.m. until A_600_ reached 0.6. Cells were pelleted at 4,000 rcf, 4 °C, for 15 minutes. Supernatant was discarded, and cells were resuspended in 2 ml TSS [l-broth containing 10% (w/v) PEG 3350, 5% (v/v) DMSO and 50 mM MgCl_2_ at pH 6.5]. T5 exonuclease DNA assembly (TEDA, [22]) reactions were incubated with 100 µl TSS competent TOP10 cells on ice for 30 min. Heat shock was performed at 42 °C for 45 s in a water bath. Cells were incubated on ice for 2 min. Briefly, 800 µl of l-broth was added, and cells were incubated on a Thermo mixer at 37 °C, 600 r.p.m., for 1 h when selecting on apramycin and 1.5 h when selecting on tetracycline. Following recovery, cells were pelleted at 10,000 rcf, resuspended in 50 µl l-broth and plated on agar plates containing antibiotic(s).

### Electroporation

*E. coli* (electroporation used only for the three-fragment TEDA cloning reaction): to make electrocompetent *E. coli* TOP10 cells, a 4-ml overnight culture was set up from a single colony and incubated at 37 °C, 220 r.p.m. The next day, the culture was diluted 1:100 in 50 ml l-broth in a 250 ml Erlenmeyer flask. The flask was incubated at 37 °C, 220 r.p.m. until A_600_ reached 0.6. Cells were pelleted at 4,000 rcf, 4 °C, for 15 min. Supernatant was discarded, and cells were then washed once with 1 ml sterile ice-cold water and once with 1 ml sterile ice-cold 10% (v/v) glycerol with pelleting steps being carried out at 10,000 rcf, 4 °C, for 2 min. Cells were finally resuspended in 500 µl sterile ice-cold 10% glycerol, thus concentrating the cells 100-fold. The desalted TEDA reaction was mixed with 50 µl competent cells in a 2-mm electroporation cuvette. Electroporation was performed at 2.5 kV with 200 Ω resistance and 25 µF capacitance. Recovery was carried out as above. To make electrocompetent *A. baumannii* AB5075 cells, a 4-ml overnight culture was set up from a single colony and grown for 16 h at 37 °C, 220 r.p.m. Cells were pelleted at 4,000 rcf, 4 °C, for 15 min. Supernatant was discarded, and cells were then washed once with 1 ml sterile ice-cold water and once with 1 ml sterile ice-cold 10% glycerol, with pelleting steps being carried out at 10,000 rcf, 4 °C, for 2 min. Cells were finally resuspended in 400 µl sterile ice-cold 10% glycerol, thus concentrating the cells 10-fold. Briefly, 25–50 ng plasmid DNA was mixed with 50 µl competent cells in a 2-mm electroporation cuvette. Electroporation and recovery were carried out as above.

### Plasmid constructions

Oligonucleotides used for priming PCRs are listed in Table S1, available in the online Supplementary Material. Plasmids were assembled from PCR products using Seamless Ligation Cloning Extract (SLiCE) or TEDA [22,23]. Plasmid inserts were confirmed by Sanger sequencing (Eurofins), and whole plasmids pAMCK14-sRNA44 and pAMCK18-Bap were sequenced by Azenta (Leipzig, Germany). To create pAMCK18-Bap*, a gBlock was ordered from IDT and used for cloning.

### RNA isolation and northern blotting

Total RNA was isolated from cells grown for 16 h in l-broth using TRIzol as described previously [24]. Northern blotting was performed using DIG-labelled riboprobes generated by *in vitro* transcription following the manual of the DIG Northern Starter Kit (Roche). Templates for *in vitro* transcriptions using T7 RNA polymerase were generated by PCR using DNA oligonucleotides listed in Table S1. Northern blotting was performed as described previously [25]. Five micrograms of total RNA was separated on a 7% urea–polyacrylamide gel in 1×Tris/borate/EDTA buffer cooled to 4 °C for each northern blot.

### Measurement of fluorescence

A single colony was inoculated in 1 ml l-broth containing apramycin and tetracycline and grown for 30 h at 37 °C and 220 r.p.m. Apart from the low volume of broth used to set up the cultures, tubes were also placed in the incubator shaker at an angle to improve aeration. This was necessary for a better production of fluorescence signal from the expressed sfGFP in *A. baumannii* AB5075. Briefly, 100 µl of the culture was then transferred into the wells of a white 96-well plate with a clear bottom (Corning, Costar), and fluorescence (arbitrary units) and absorbance at 600 nm (A_600_) were measured in a BioTek Synergy HTX Multimode Reader. Fluorescence was measured with a top read, with excitation filter 485/20, emission filter 528/20 and gain 45. The fluorescence of different strains was compared by quantifying the fluorescence observed relative to A_600_ (F/A_600_). l-agar plates were imaged using epi blue light and a 510DF10 filter in the ImageQuant LAS4000 (GE Healthcare).

## Results

### Construction of plasmids to study sRNA–mRNA interactions

**Fig. 1.**
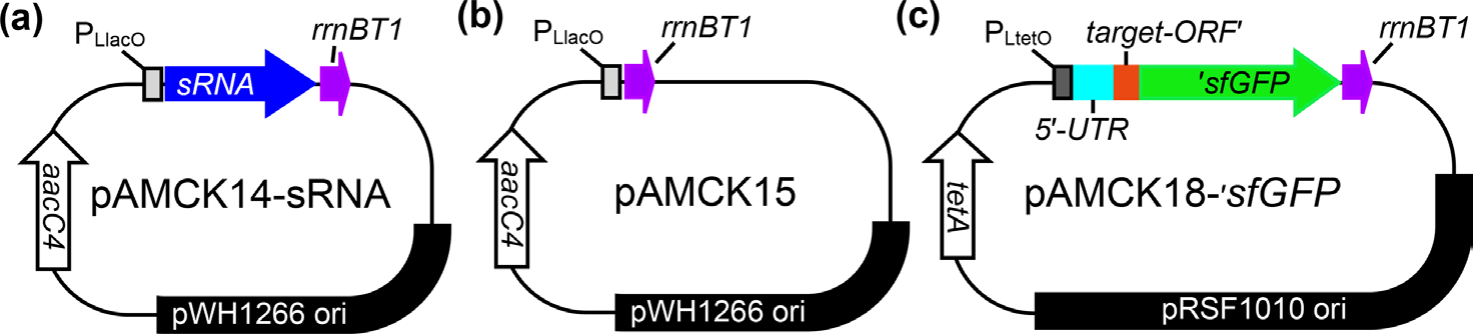
Schematic plasmid maps of (**a**) pAMCK14, (**b**) pAMCK15 and (**c**) pAMCK18. (**a**) The plasmid pAMCK14 expresses a small RNA from the P_LlacO_ promoter, which is terminated by the rrnBT1 terminator. (**b**) In pAMCK15, the same P_LlacO_ promoter is terminated almost immediately and expresses a small, nonsense RNA, which is identical with the nonsense RNA expressed from pJV300 [7]. Both plasmids possess the pWH1266 origin of replication and the *aacC4* gene from pMHL-2, conferring resistance to apramycin [27]. (**c**) Plasmid pAMCK18 expresses a translational fusion of a target gene fused to a start-codon-less sfGFP obtained from pXG10sf [11]. The fusion protein is expressed from the P_LtetO_ promoter and terminated by the rrnBT1 terminator as in the original pXG10 plasmid [7,11]. Plasmid pAMCK18 harbours the *tetA* gene, conferring tetracycline resistance, and the pRSF1010 origin of replication. The plasmid schematics are not drawn to scale.

The original two-plasmid system comprises the pP_L_ plasmid, which carries the sRNA gene, and the pXG10/pXG10sf plasmid, which carries the target mRNA-*GFP* fusion [7,11]. These plasmids contain origin of replications (ColE1 and pSC101*) and selection genes (resistance to ampicillin and chloramphenicol) that are unsuitable for use in multidrug-resistant *A. baumannii* strains, such as the widely used strain AB5075 [26]. To adapt the system for *A. baumannii* (Fig. 1), the sRNA and mRNA expression regions of the original two-plasmid system were retained, and origins of replication and resistance cassettes suitable for *A. baumannii* were introduced. To construct the sRNA expression plasmid (pAMCK14, Fig. 1), a fragment of the pP_L_ plasmid containing the P_LlacO_-driven sRNA expression region was fused with the *rrnBT1* terminator and the ColE1 origin of replication and combined with a fragment of the pAMCK2 plasmid carrying the widely used pWH1266 origin of replication and the apramycin resistance gene, *aacC4*, from pMHL2 [15,27]. The ColE1 origin of replication enables replication in *E. coli*, while the pWH1266 origin of replication allows replication in *A. baumannii*, making the resulting plasmid a shuttle vector. To construct the control plasmid (pAMCK15, Fig. 1), the sRNA gene was deleted from pAMCK14 while retaining the *rrnB1* terminator. This plasmid is equivalent to the pJV300 control plasmid used in the original two-plasmid system and would express a short nonsense RNA [7]. To construct the target mRNA expression plasmid, a fragment from pXG10sf containing the P_LtetO_-driven target mRNA region fused to the start codon-lacking *sfGFP*, an *rrnBT1* terminator and the pSC101 origin of replication was combined with a fragment containing the pRSF1010 origin of replication, which was obtained from *S. enterica* serovar Typhimurium SL1344 [24], and a fragment of the pBR322 plasmid carrying the tetracycline resistance gene *tetA*. The resulting 10.3 kb plasmid contains both the pSC101 and pRSF1010 origins of replication and presents a shuttle vector capable of replication in both *E. coli* and *A. baumannii* AB5075. Although fluorescent, mutations in *sfGFP* were detected upon sequencing, which were corrected. Because the pRSF101 origin of replication works in *A. baumannii* and *E. coli*, the pSC101 origin of replication was removed to construct the final plasmid (pAMCK18, Fig. 1), which retains only the P_LtetO_-driven target mRNA expression region fused to *sfGFP* and terminated by *rrnBT1*. This ~8 kb pAMCK18 plasmid is also a shuttle vector capable of replication in both *E. coli* and *A. baumannii*, as expected from the broad range pRSF1010 origin of replication. The retention of the sRNA and target mRNA expression regions of the original two-plasmid system ensured that the primers used to amplify the backbones and inserts for cloning could be used with the original and new two-plasmid systems. All plasmids used in this study are listed in Table 1.

**Table 1.** Plasmids used in this study

Plasmid name	Description	Reference
pAMCK14-Aar	Expression of Aar from P_LlacO_ promoter. Origin of replication ori_pWH1266_. ApraR	This study
pAMCK14-sRNA44	Expression of sRNA44 from P_LlacO_ promoter. Origin of replication ori_pWH1266_. ApraR	This study
pAMCK14-sRNA44*	Expression of sRNA44* from P_LlacO_ promoter. Origin of replication ori_pWH1266_. ApraR	This study
pAMCK18-CarO	Expression of CarO-sfGFP fusion protein from P_LtetO_ promoter. Origin of replication ori_pRSF1010_. TetR	This study
pAMCK18-Bap	Expression of Bap-sfGFP fusion protein from P_LtetO_ promoter. Origin of replication ori_pRSF1010_. TetR	This study
pAMCK18-Bap*	Expression of Bap*-sfGFP fusion protein from P_LtetO_ promoter. Origin of replication ori_pRSF1010_. TetR	This study
pP_L_-Aar	Expression of Aar from P_LlacO_ promoter. Origin of replication ori_ColE1_. AmpR	[28]
pP_L_-Aar*	Expression of Aar* from P_LlacO_ promoter. Origin of replication ori_ColE1_. AmpR	[28]
pP_L_-sRNA44	Expression of sRNA44 from P_LlacO_ promoter. Origin of replication ori_ColE1_. AmpR	This study
pXG10sf-CarO	Expression of CarO-sfGFP fusion protein from P_LtetO_ promoter. Origin of replication ori_pSC101*_. CmR	[11,28]
pXG10sf-Bap	Origin of replication ori_pSC101*_. CmR	This study
pAMCK15	Expression of nonsense RNA from P_LlacO_ promoter. Origin of replication ori_pWH1266_. ApraR	This study
pJV300	Expression of nonsense RNA from P_LlacO_ promoter. Origin of replication ori_ColE1_. AmpR	[7]

### Validation of the Aar-*carO* translational repression in *E. coli* TOP10 and *A. baumannii* AB5075

**Fig. 2.**
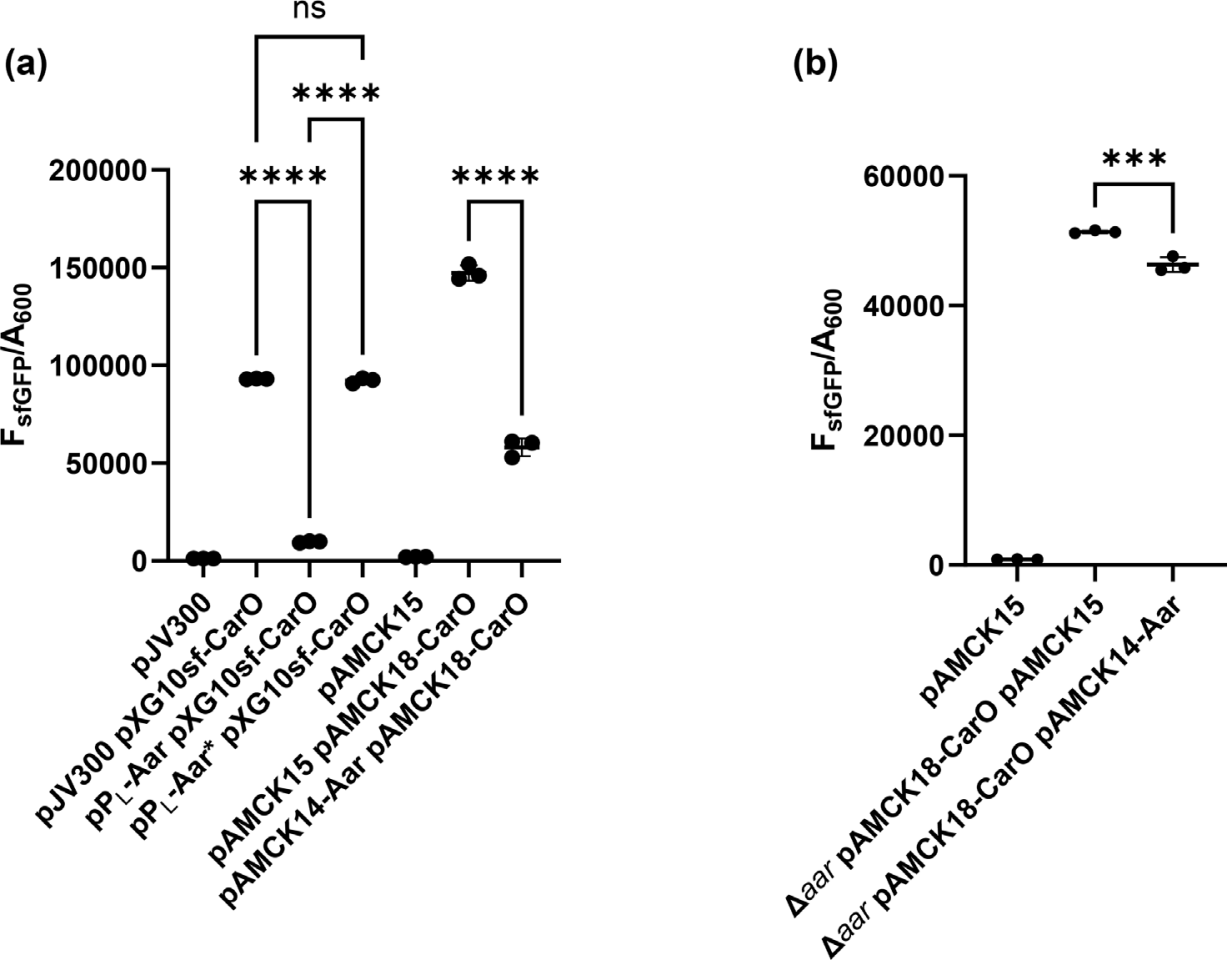
The small RNA Aar represses translation of CarO′-sfGFP in (**a**) *E. coli* TOP10 and (**b**) *A. baumannii* AB5075. Comparison of the original two-plasmid system consisting of pJV300, pPL and pXG10sf plasmids and newly developed pAMCK14, pAMCK15 and pAMCK18 system showing that expression of Aar represses translation of CarO′-sfGFP, validating earlier findings [28]. The data are expressed as fluorescence (F_sfGFP_) divided by culture absorbance (A_600_) after 30 h. Three independent experiments were performed per strain. Whether differences are statistically significant was assessed by one-way ANOVA with Tukey’s multiple comparisons test. *** indicates *P*_adj_<0.001, **** indicates *P*_adj_<0.0001 and ns indicates *P*_adj_>0.05.

**Fig. 3.**
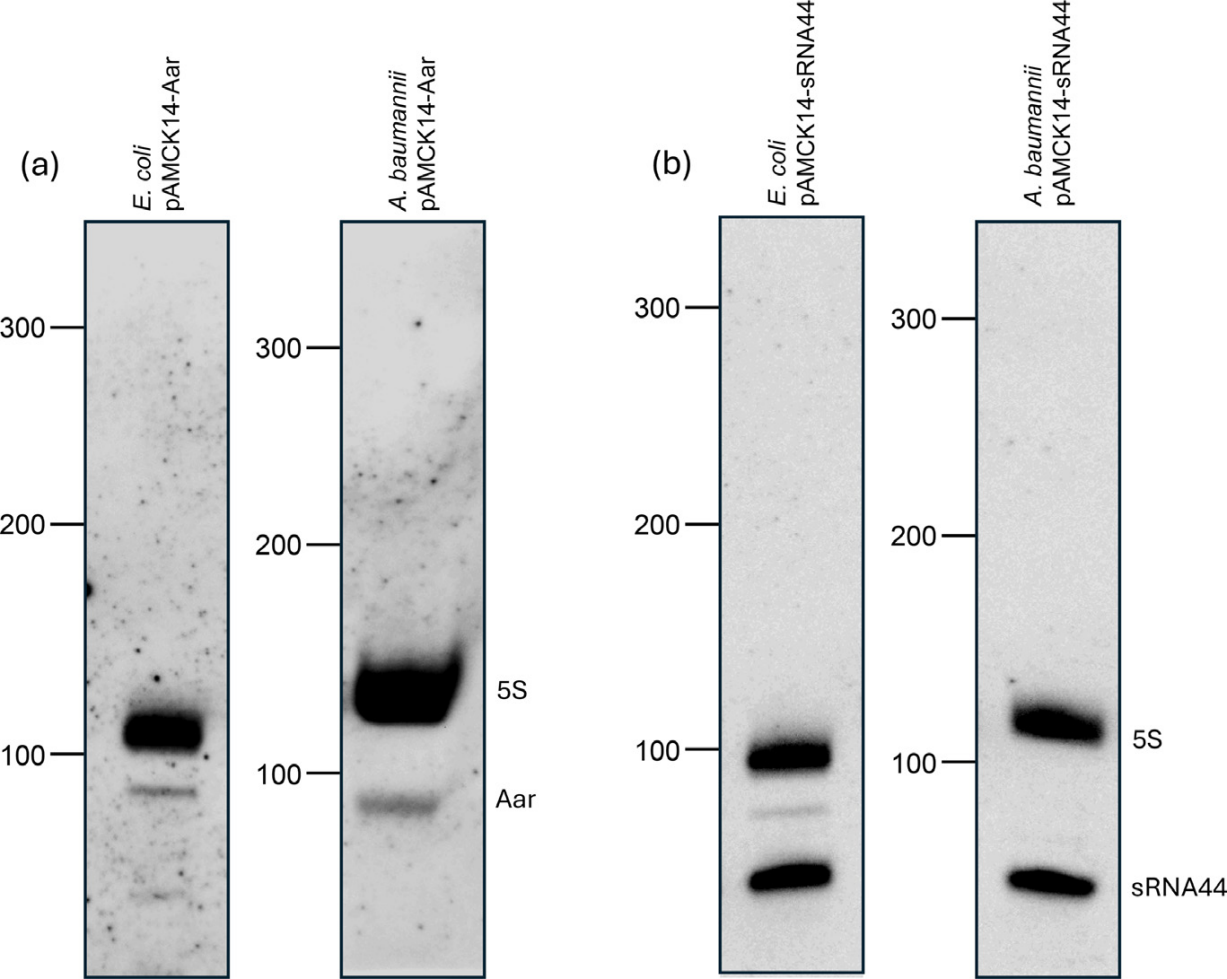
Expression of Aar and sRNA44 from pAMCK14 is similar in *E. coli* and *A. baumannii*. Five µg of total RNA was subjected to northern blotting using DIG-labelled riboprobes to detect transcripts of (**a**) Aar and (**b**) sRNA44. A riboprobe specific for 5S rRNA of each species was used as a loading control.

To evaluate the functionality of the new two-plasmid system in *A. baumannii* AB5075 and *E. coli* TOP10, translational repression of the *carO* mRNA by the sRNA Aar of *A. baumannii* AB5075 was assessed. The interaction was previously validated in *E. coli* using the original two-plasmid system, where expression of Aar from the pP_L_ plasmid reduced fluorescence from the pXG10-CarO-sfGFP reporter compared to the pJV300 control; however, validation of the interaction in *A. baumannii* AB5075 previously required a more complex strategy relying upon the introduction of several chromosomal mutations [28]. Identical sequences of Aar and *carO* used in the original two-plasmid system were cloned into pAMCK14 (resulting in pAMCK14-Aar expressing Aar) and pAMCK18 (resulting in pAMCK18-CarO expressing the CarO-sfGFP fusion protein). As a control, pAMCK18-CarO was paired with the control plasmid pAMCK15. Plasmid combinations were measured for fluorescence in *E. coli* TOP10 and *A. baumannii* AB5075 (Δ*aar* when containing both plasmids) and normalized to culture absorbance (Fig. 2). In *E. coli*, the new system largely recapitulated the findings from the original two-plasmid system (Fig. 2a). In *E. coli*, the level of fluorescence of the pAMCK18-CarO fusion was ~1.5-fold higher than the pXG10sf-CarO variant, possibly due to the corrected mutations in *sfGFP*. Co-expression of wild-type Aar reduced translation of *carO* mRNA by ~77%, comparable to ~90% reduction observed with the original system (pXG10sf/pP_L_/pJV300), showing that the new two-plasmid system is functionally equivalent to the original system. The level of fluorescence in *A. baumannii* AB5075 Δ*aar* pAMCK18-CarO expressing wild-type Aar from pAMCK14 was reduced (10%, *P*=0.0002) compared to the strain harbouring the control plasmid pAMCK15, validating translational repression of CarO-sfGFP in *A. baumannii* AB5075 (Fig. 2b, [28]). Consistent with previous observations [28], the repression of *carO* translation by Aar is much weaker in *A. baumannii* compared to *E. coli*. The weaker effect is not caused by lower expression of Aar from pAMCK14, because expression in * E. coli* and *A. baumannii* is similar as judged by northern blotting (Fig. 3a), suggesting that there are additional endogenous regulatory factors influencing the level of *carO* translation using the two-plasmid system in *A. baumannii*. Taken together, the data suggest that the new two-plasmid system enables the study of sRNA-mediated post-transcriptional regulation in *E. coli* and *A. baumannii*.

### Translation of *bap* is inhibited by sRNA44

**Fig. 4.**
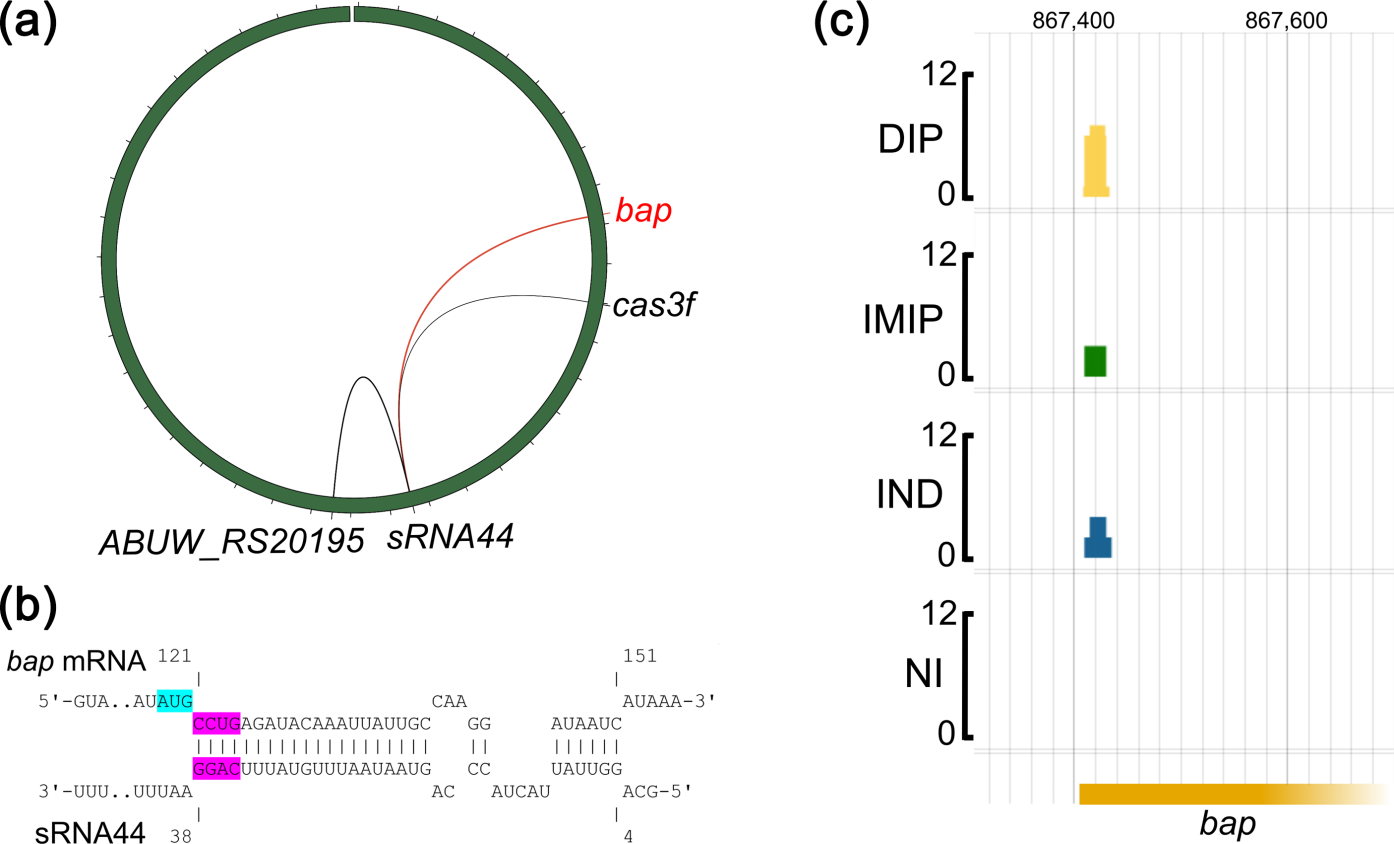
(a) Three mRNAs (*bap*, *ABUW_RS20195* and *cas3f*) were ligated to sRNA44 in a Hi-GRIL-seq experiment [28]. The green circle represents the chromosome of *A. baumannii* AB5075 with the origin of replication at the top of the circle, and the location of *sRNA44*, *bap*, *ABUW_RS20195* and *cas3f* is highlighted. The width of the lines is proportional to the number of chimeric sequencing reads obtained from the Hi-GRIL-seq experiment. (b) Predicted interactions of *bap* mRNA (top sequence) with sRNA44 (bottom sequence) using IntaRNA [31,59]. The *bap* start codon is highlighted in light blue. The nucleotides 35-38 in sRNA44 were mutated from 5′-CAGG-3′ to 5′-GACC-3′ to create sRNA44* (highlighted in pink with their base-pairing nucleotides in *bap* mRNA). The numbers indicate positions relative to the transcriptional start sites. (c) Mapped Hi-GRIL-seq-derived chimeric reads consisting of bap mRNA and sRNA44. The scale is 0–12 reads. The numbers at the top of the figure indicate the genomic location. The *bap* gene is truncated for illustration purposes. IND=T4 RNA ligase induced, IMIP=T4 RNA ligase induced with additional brief ‘shock’ with sub-inhibitory concentration of imipenem, DIP=T4 RNA ligase induced with additional brief limitation of iron by the addition of 2,2′-dipyridyl, NI=control, T4 RNA ligase not induced.

**Fig. 5.**
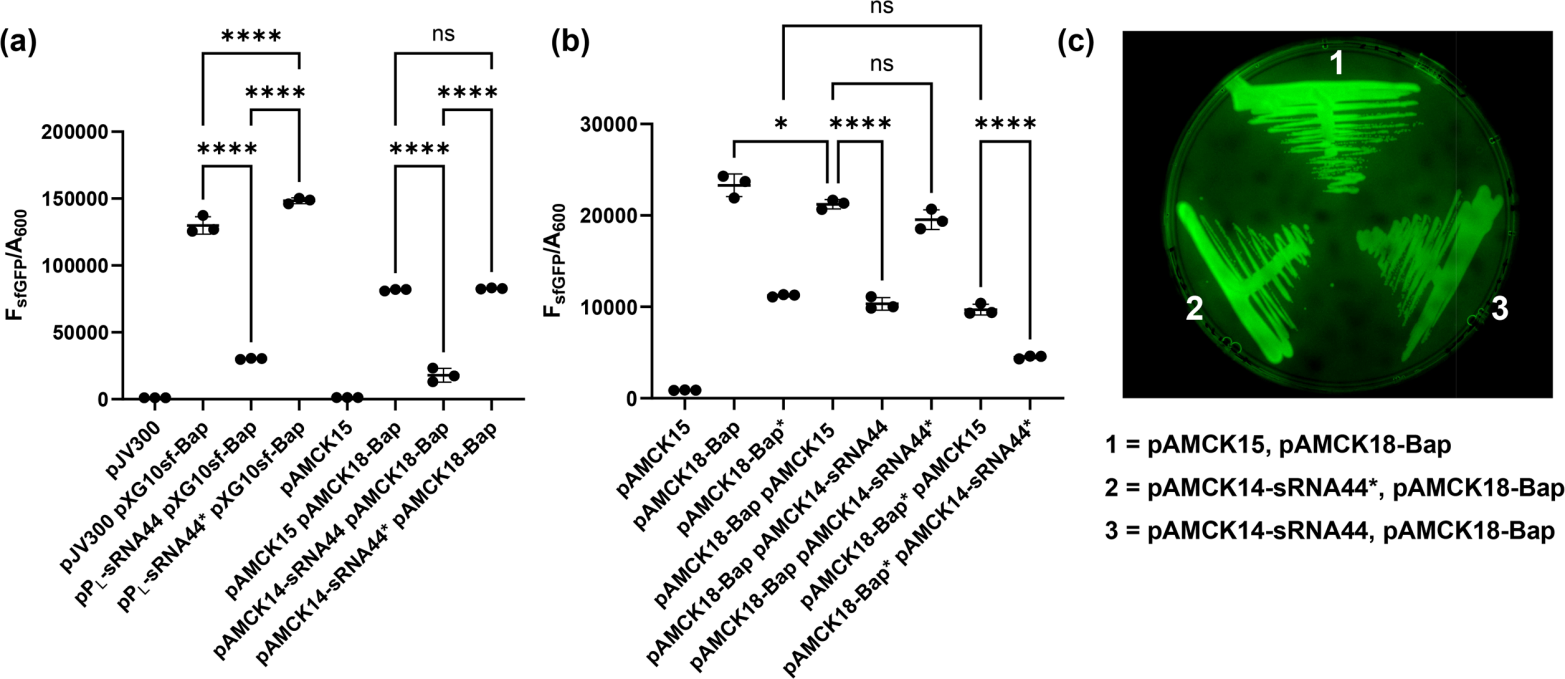
The small RNA sRNA44 represses translation of Bap′-sfGFP in (**a**) *E. coli* TOP10 and (**b**) and (c) *A. baumannii* AB5075 WT. (**a) and (b**) The data are expressed as fluorescence (F_sfGFP_) divided by culture absorbance (A_600_) after 30 h. Three independent experiments were performed per strain. Whether differences are statistically significant was assessed by one-way ANOVA with Tukey’s multiple comparisons test. **** indicates *P*_adj_<0.0001 and ns indicates *P*_adj_>0.05. In (**c**), *A. baumannii* AB5075 carrying the indicated plasmids was streaked on an l-agar plate containing apramycin and tetracycline and grown for 16 h before the plate was imaged.

To test a novel, unvalidated sRNA–mRNA interaction of *A. baumannii*, a putative sRNA–mRNA pair was selected that had been previously identified by Hi-GRIL-seq [28]. Biofilm formation is a key virulence trait of *A. baumannii* virulence and persistence in hospital environments, and the biofilm-associated protein Bap is involved in biofilm formation and is widely present in *Acinetobacter* spp. [29,30]. Hi-GRIL-seq recovered 28 chimeric sequencing reads ligated between sRNA44 and *bap* mRNA, suggesting that sRNA44 may regulate the translation of *bap* mRNA through base-pairing [28]. Two additional mRNAs were ligated to sRNA44 encoding a hypothetical gene (*ABUW_RS05340*, 22 chimeric reads) and the type-I-F CRISPR-associated helicase Cas3 (11 chimeric reads, Fig. 4a, [28]). The *bap* mRNA was prioritized because sRNA44–*bap* mRNA chimeric reads were most frequent, and IntaRNA predicted the formation of an RNA duplex between sRNA44 and *bap* mRNA just downstream of the start codon of the *bap* mRNA (Fig. 4b, [31]). Chimeric reads from all Hi-GRIL-seq conditions mapped to the AB5075 genome (IND=T4 RNA ligase induced, IMIP=T4 RNA ligase induced with additional brief ‘shock’ with sub-inhibitory concentration of imipenem, DIP=T4 RNA ligase induced with additional brief limitation of iron by the addition of 2,2′-dipyridyl, NI=control, T4 RNA ligase not induced) matched the predicted duplex location (Fig. 4c). The Hi-GRIL-seq data for sRNA44 can be accessed and browsed online using Jbrowse2: https://bioinf.gen.tcd.ie/hi-gril-seq/sRNA44 [32]. To test the interaction, plasmids pAMCK14-sRNA44 (expressing sRNA44) and pAMCK18-Bap (expressing the *bap* 5′ UTR fused to sfGFP, including the predicted duplex region and the first 13 codons of *bap*) were constructed (Fig. 4c). The transcriptional start sites for sRNA44 and *bap* were previously defined by differential RNA-seq [25]. Expression of sRNA44 from the pAMCK14 plasmid was similar in *E. coli* and *A. baumannii* (Fig. 3b). For comparison, the original two-plasmid system was constructed with sRNA44 expressed from pP_L_-sRNA44 and the Bap-sfGFP fusion protein expressed from pXG10sf-Bap. To disrupt and restore the interaction of sRNA44 with *bap* mRNA, nucleotides 35–38 of sRNA44 were mutated from 5′-CAGG-3′ to 5′-GACC-3′ in pPL-sRNA44 to construct pPL-sRNA44* and in pAMCK14 to construct pAMCK14-sRNA44*, and complementary mutations were introduced in pAMCK18-Bap* (Fig. 4b). RNA structure predictions of sRNA44 and sRNA44* showed that the mutated nucleotides are located in a single-stranded region and did not interfere with their structure (Fig. S1). Plasmid combinations were again measured for fluorescence as before and normalized on culture absorbance. Using the original system in *E. coli*, expression of sRNA44 from pP_L_-sRNA44 led to a reduction of fluorescence by 77% compared to the strain carrying the pJV300 control plasmid, indicating that sRNA44 represses production of the Bap-sfGFP fusion protein (Fig. 5a). Introduction of the three-point mutations of sRNA44* relieved the repression, and Bap-sfGFP abundance was even slightly higher than the strain containing the pJV300 control plasmid. In both *E. coli* TOP10 (78% reduction of fluorescence, Fig. 5a) and *A. baumannii* AB5075 (51% reduction of fluorescence, Fig. 5b, c), co-expressing pAMCK14-sRNA44 along with pAMCK18-Bap resulted in a decrease in fluorescence signal compared to the control plasmid pAMCK15. The plasmid pAMCK18-Bap* expressing mutated Bap*-sfGFP showed lower fluorescence compared to pAMCK18-Bap, suggesting that translation of Bap*-sfGFP might be impaired by the introduction of SNPs near the RBS (Fig. 5b). However, fluorescence was well above the strain carrying pAMCK15. Co-expressing pAMCK14-sRNA44* with pAMCK18-Bap* resulted in a 46% reduction of fluorescence compared to the control strain carrying pAMCK15 and pAMCK18-Bap*, indicating that the introduction of complementary point mutations restored the interaction of sRNA44* and Bap*-sfGFP (Fig. 5b).

In contrast to the sfGFP fusions with CarO, the Bap-sfGFP abundance was higher in the pXG10sf-Bap compared to pAMCK18-Bap in *E. coli* (Figs2a  5a). In both bacteria, co-expressing the mutant variant sRNA44* from pP_L_-sRNA44* or pAMCK14-sRNA44* along with pAMCK18-Bap increased fluorescence was observed compared to the strains expressing wild-type sRNA44, and rescue of fluorescence production in *A. baumannii* expressing sRNA44* with Bap*-sfGFP suggests that the nucleotides 35–38 of sRNA44 are required for base-pairing with *bap* mRNA (Fig. 5b).

## Discussion

Small regulatory RNAs which base-pair with a target RNA molecule are found in all kingdoms of life and provide an additional layer of gene regulation. In bacteria, foundational mechanistic understanding of these interactions was first generated in * E. coli* and *S. enterica* by using genetic tools designed for these model organisms. One versatile and highly utilized genetic tool to study the mechanisms of sRNA-mediated post-transcriptional regulation consists of two plasmids, of which one expresses a small RNA and the other the target RNA fused to a reporter gene, first GFP and later sfGFP [7,11, 33,43]. This general approach has since been adopted for other bacteria, such as *Vibrio cholerae* and *Klebsiella pneumoniae* [44,47], and used to probe sRNA–mRNA interactions in *Bacteroides thetaiotaomicron* and *A. baumannii* using *E. coli* as a heterologous host [28,48]. The key advantages of this system are that the sRNA and mRNA are expressed from separate plasmids, enabling clean and rapid validation of *trans* interactions, and that plasmids are easily constructed and quickly mixed and matched. In this study, the original two-plasmid system was extended to function in multidrug-resistant *A. baumannii* by swapping origins of replication with ones that support stable replication in *A. baumannii* and suitable selection genes. Both plasmids still contain origins of replication that function in *E. coli* to allow the study of RNA–RNA interactions in a heterologous host. We note that fluorescence in *E. coli* is ~threefold to fourfold higher from the same pAMCK18-CarO/Bap plasmids compared to *A. baumannii*, which could be due to a lower transcription or plasmid copy number in *A. baumannii*. Alternatively, host-specific differences in the availability or function of RNA-binding proteins, such as the notable differences of Hfq between *E. coli* and *A. baumannii* [49,51], may modulate the stability or formation of sRNA–mRNA duplexes in the native versus heterologous background. Nevertheless, we validated the translational repression of CarO by the sRNA Aar in *A. baumannii* [28] and validated a new sRNA-mediated regulation of an *A. baumannii* virulence factor, the Bap protein [29,30]. The strain-specific differences for the Aar–carO mRNA interaction highlight the need for a two-plasmid system in *A. baumannii* to catch biological effects in the homologous host that might be missed in a heterologous system. The new system allows for a nuanced study of RNA–RNA interactions in *A. baumannii* to explore endogenous regulatory mechanisms. Future experiments will be required if the translational repression of Bap by sRNA44 has physiological consequences such as the level of biofilm formation or cellular adhesion. Beyond validating native regulatory interactions, the newly constructed two-plasmid system may also be used to construct and benchmark synthetic small RNAs in *A. baumannii*, similar to toolboxes created for *E. coli* [52,53].

Although the utility of the pAMCK two-plasmid system in *A. baumannii* is clear, a few limitations and challenges should be acknowledged. Because of the use of two multi-copy plasmids and constitutive promoters, the stoichiometry of sRNA–mRNA interactions and associated effects on cellular physiology will be different from wild-type cells, which necessitates follow-up experiments to validate biological relevance. Differences in fluorescence produced by protein fusions are not easily anticipated due to the inherent individual folding kinetics of every protein under assessment, and host-specific variation in translation initiation of target protein-GFP fusions may also limit the detectability of fluorescence. In cases where the signals fall below the detection limit, sfGFP abundance can still be measured by Western blotting using GFP-specific antibodies, though this is more labour-intensive. Because sRNAs and target genes must be cloned at nucleotide resolution, sequence and ligation–independent cloning (SLIC), SLiCE, TEDA, Gibson assembly or similar methods of cloning are required. Because this involves PCR-based amplification of the plasmid backbones, we recommend whole plasmid sequencing after cloning, which multiple sequencing providers offer at low cost. To reduce the likelihood of recombination of plasmid sequences with the chromosome in *A. baumannii* and to be able to stably recover the plasmids from bacterial cultures stored long-term, the two-plasmid system could be used and stored in a Δ*recA* background as suggested for *E. coli* [7] in the future. However, the original two-plasmid system was used reliably in wild-type *S. enterica* and *V. cholerae*, and we did not have indications of where the plasmids were chromosomally inserted [11,44]. It is anticipated that the system should also work in non-*baumannii Acinetobacter* spp., such as *Acinetobacter baylyi*, *Acinetobacter pittii* or *A. calcoaceticus*, because these organisms support replication of pWH1266 [15,54, 55]. The plasmid pAMCK18 should possess a much broader host range using the pRSF1010 origin of replication, as plasmids based on pRSF1010 have been used in numerous bacteria, including *Pseudomonas* spp. [56], *Pasteurella multocida* [57] and even cyanobacteria [58]. To create a species-specific two-plasmid system for any pRSF1010-replicating bacteria, it would only require another co-replicating plasmid. Finally, although P_LlacO_ and P_LtetO_ are theoretically inducible in strains containing *lacI* and *tetR*, such regulatory elements are not yet standard in *A. baumannii*. Incorporation of *lacI* and *tetR* into the host would broaden the utility of the system when investigating regulatory effects following induction of sRNAs [10].

In conclusion, the newly developed shuttle pAMCK two-plasmid system enables robust validation of sRNA–mRNA interactions and facilitates mechanistic dissection of the interactions through targeted mutagenesis of predicted base-pairing regions in both *A. baumannii* and *E. coli*.

## Supplementary material

10.1099/mic.0.001639 Uncited Supplementary Material 1.

## Abbreviations

SLiCE: Seamless Ligation Cloning Extract
sRNAs: small regulatory RNAs
TEDA: T5 exonuclease DNA assembly
TSS: transformation and storage solution
