# Supplementary material for: Development of two compatible plasmids to assess sRNA-mediated post-transcriptional regulation in Acinetobacter baumannii
Source: Microbiology (Reading). 2025 Nov 25;171(11):001639. doi: 10.1099/mic.0.001639 (PMC12646401; doi:10.1099/mic.0.001639)
Supplement: Uncited Supplementary Material 1. [file mic-171-01639-s001.pdf]

SUPPLEMENTARY DATA

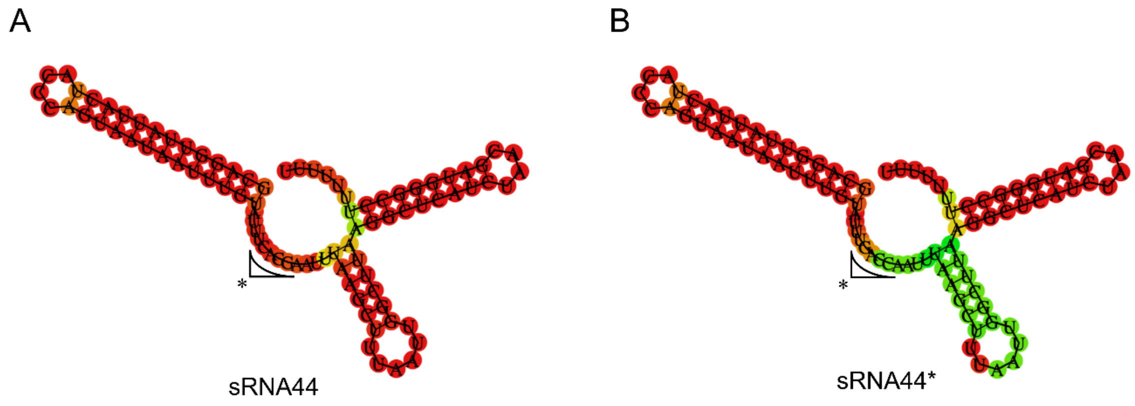

Figure S1. Structure predictions of sRNA44 **(A)** and sRNA44* **(B)**. The prediction was carried out using RNAfold [55]. The positions of the mutated nucleotides are highlighted with an asterisk.

Table S1. List of DNA oligonucleotides used in this study.

No.	Purpose	Sequence (5′-3′)
1438	Amplification of sRNA44 insert	GTGAGCGGATAACAAGATACTGAGCACGC AGGTTATTACTACCCAG
1439	Amplification of sRNA44 insert	GCCTTTCGTTTTATTTGATGCCTCTAGACAC CTATTATTATCAAGTGGC
143	Amplification pXG10sf backbone	GCTAGCGGATCCGCTGGCTCCGCTGC
134	Amplification pXG10sf backbone	ATGCATGTGCTCAGTATCTCTATCAC
141	Amplification pP_L backbone	GTGCTCAGTATCTTGTTATCCGCTCAC
142	Amplification pP_L backbone	TCTAGAGGCATCAAATAAAACGAAAGGC
1436	Amplification of bap insert	ACTGAGCACATGCATGTAAAAATGATCACA TTAC
1437	Amplification of bap insert	AGCGGATCCGCTAGCTTTATGATTATCCTTG GC
2517	Construction of pAMCK14 (pPL-sRNA44-AbOri-aacC4)	GGACTTATCATCCAACCTGTCTGTCAGACC AAGTTTACG
1958	Construction of pAMCK14 (pPL-sRNA44-AbOri-aacC4)	TTTCCCCGAAAAGTGCCACCTG
2518	Construction of pAMCK14 (pPL-sRNA44-AbOri-aacC4)	CGTAAACTTGGTCTGACAGACAGGTTGGAT GATAAGTCC
2519	Construction of pAMCK14 (pPL-sRNA44-AbOri-aacC4)	TAATGGTTTCTTAGACGTCAGGTGGCACTTT TCGGGGAAAGATCGTAGAAATATCTATG
2520	Construction of pAMCK12 (pXG10-bap-pRSF1010-ori-tetA)	AGAACATATCCATCGCGTCCGCCATCTCCA GCAGCCACGTCTCATTTTCGCCAGATATC
2521	Construction of pAMCK12 (pXG10-bap-pRSF1010-ori-tetA)	GCGATAGACTGTATGTAAACATTTGATATC GAGCTCGCTTGG
2522	Construction of pAMCK12 (pXG10-bap-pRSF1010-ori-tetA)	CCAAGCGAGCTCGATATCAAATGTTTACAT ACAGTCTATCGC
2523	Construction of pAMCK12 (pXG10-bap-pRSF1010-ori-tetA)	AAAGCTTATCGATGATAAGCTGTCAAACAT GAGAAAGAAGGCCATCCTGACGGATGGCC
1560	Construction of pAMCK12 (pXG10-bap-pRSF1010-ori-tetA)	TTCTCATGTTTGACAGCTTATCATCG
1454	Construction of pAMCK12 (pXG10-bap-pRSF1010-ori-tetA)	GGCTGCTGGAGATGGCGGAC
2572	Construction of pAMCK15 control (removal of sRNA44 from pAMCK14)	ATACTGAGCACTCTAGAGGCATCAAATAAA ACG
2573	Construction of pAMCK15 control (removal of sRNA44 from pAMCK14)	GCCTCTAGAGTGCTCAGTATCTTGTTATCCG C
2574	Repair of mutations in sfgfp gene that originated from pXG10 bap in pAMCK12	CCAACACTTGTCACTACTCTCACTTATGGTG
2575	Repair of mutations in sfgfp gene that originated from pXG10 bap in pAMCK12	TTGGTGAGAATATTTGTCCTACTCAGGAGA GCGTTCACCG

2576	Repair of mutations in sfgfp gene that originated from pXG10 bap in pAMCK12	GACAAATATTCTCACCAATAAAAAACGCCCGGC
2577	Repair of mutations in sfgfp gene that originated from pXG10 bap in pAMCK12	AGTAGTGACAAGTGTTGGCCATGGAACAGGTAG
2578	Repair of mutations in sfgfp gene that originated from pXG10 bap in pAMCK12	GAGTAGGACAAATCCGCCGCCCTAGACCTAGGGTACGGG
2577	Repair of mutations in sfgfp gene that originated from pXG10 bap in pAMCK12	AGTAGTGACAAGTGTTGGCCATGGAACAGGTAG
1095	Forward primer; template PCR for *in vitro* transcription of Aar (sRNA21) riboprobe	GTAGGTTGATATGAACCTCACG
1096	Reverse primer; template PCR for *in vitro* transcription of Aar (sRNA21) riboprobe	GAATTAATACGACTCACTATAAAAAAAATACGCAATGATTGGG
183	Forward primer; template PCR for *in vitro* transcription of sRNA44 riboprobe	GCAGGTTATTACTACCCAG
185	Reverse primer; template PCR for *in vitro* transcription of sRNA44 riboprobe	GAATTAATACGACTCACTATAAGCACATAAAAAAAGGCCC
2468	Forward primer; template PCR for *in vitro* transcription of 5S rRNA riboprobe (*E. coli*)	TGGTCCCACCTGACCCCATG
2469	Reverse primer; template PCR for *in vitro* transcription of 5S rRNA riboprobe (*E. coli*)	GAATTAATACGACTCACTATAGTTCCCTACTCTCGCATGG
622	Forward primer; template PCR for *in vitro* transcription of 5S rRNA riboprobe (*A. baumannii*)	GCTGGCGACCATAGCAAG
623	Reverse primer; template PCR for *in vitro* transcription of 5S rRNA riboprobe (*A. baumannii*)	GAATTAATACGACTCACTATAATGACTTACTCTCACATGG
